# Surface Mould Brachytherapy for Skin Cancers: The British Columbia Cancer Experience

**DOI:** 10.7759/cureus.6412

**Published:** 2019-12-18

**Authors:** Stephanie Casey, Joseph Awotwi-Pratt, Gaurav Bahl

**Affiliations:** 1 Radiation Oncology, British Columbia Cancer Agency, Abbotsford, CAN; 2 Medical Physics, British Columbia Cancer Agency, Abbotsford, CAN

**Keywords:** surface mould brachytherapy, non melanoma skin cancer, : basal cell carcinoma, squamous cell carcinoma, radiotherapy

## Abstract

Purpose

To report on skin tumor treatment with surface mould brachytherapy at our institution.

Methods

This was a retrospective review for all patients with skin tumors treated using Ir-192 high dose rate (HDR) surface mould brachytherapy from January 1, 2010 to December 31, 2017 in British Columbia. We identified 65 lesions (59 patients). Median age at diagnosis was 83 (range = 45-97). The majority were basal cell (54%, n = 35) or squamous cell carcinomas (31%, n = 20). Most lesions were located in the head and neck region. The most commonly used RT dose was 40 Gy/10 fractions; all patients had individualized CT-based planning.

Results

The two-year overall survival (OS) was 77.6% and two-year progression-free survival (PFS) was 71.5%. Most deaths were from unrelated causes. Response was assessed in clinic 2-4 months post-treatment. Our complete response (CR) rate was 96.8%, with partial response in two patients; two patients could not be assessed for response. We report a two-year local control (LC) rate of 84.9%, and local recurrence in five patients. The procedure was well tolerated, with no grade 3-5 acute or late toxicities. There was one case of grade 2 radionecrosis (Common Terminology Criteria for Adverse Events (CTCAE) v. 4.03). The 100% isodose line median depth was 0.5 cm, and median surface dose = 126.5%. The median V_90_ = 92.3%.

Conclusion

Surface mould brachytherapy for skin tumors is a safe and effective modality, with excellent response rates. It is well-tolerated and a non-invasive option for elderly patients with comorbidities.

## Introduction

The incidence of skin malignancies is on the rise, with a recent estimate of over 3 million people in the US [1-4]. Of these increasingly common cancers, basal cell carcinomas (BCC) followed by squamous cell carcinomas (SCC) constitute the majority [5]. These tumors have low metastatic potential, but can be locally destructive, resulting in significant morbidity and disfigurement [6].

A plethora of options are available for treating skin malignancies, including: topical therapy (such as Imiquimod), cryotherapy, curettage, surgical resection and radiotherapy (both external beam techniques as well as brachytherapy). Surgery is most frequently used, given the low overall treatment time and excellent local control (LC) rates - generally 95%. The highest overall cure rates are seen with Mohs microsurgery, at 95-99% LC [5, 7-11]. However, there remain scenarios where surgery is not feasible, either because of tumor concerns (size, residual disease, perineural invasion), concerns regarding cosmetic outcome or functional status post-surgery (Ex. Tumors on the ears, nose, eyelids) or patient factors (elderly patients, or those with comorbidities which preclude them from surgical resection) [2, 5, 8, 10]. In recent years, technological advances have improved the ability to deliver safe and effective radiotherapy, resulting in renewed interest in this modality [2, 5, 12, 13]. Brachytherapy techniques carry many advantages: they can deposit a significantly higher dose within a tumor, with better sparing of adjacent normal structures versus plans using external beam radiotherapy (EBRT) or electron therapy, which sometimes require irradiating a large volume of tissue in order to adequately cover the malignancy, increasing the potential for toxicity. Brachytherapy is best for lesions located at, or just below the skin surface, given the dose is substantially decreased by 5-mm depth from the skin surface - lesions deeper than 5 mm are better covered by interstitial techniques [3-5, 14, 15]. High-dose rate (HDR) brachytherapy is individualized, very conformal, results in higher biologically equivalent doses, and has also shown high local control (LC) rates (83-100%) [3, 5, 6, 16-18]. Furthermore, recent advances in planning software and algorithms are likely to improve the cosmetic and functional outcomes compared with EBRT and surgery, though currently little has been published in the way of large trials [3, 4, 10, 14, 16-19].

The purpose of our paper is to add to the body of knowledge regarding the use of surface brachytherapy for the treatment of skin malignancies. We report on our institution’s experience with using 192Ir-based HDR surface mould brachytherapy for the treatment of skin malignancies.

## Materials and methods

A retrospective review of all patients treated with surface mould 192Ir-based HDR brachytherapy at our center from January 1, 2010 to December 31, 2017 was performed. Approval for this study was obtained from the Institutional Research Ethics Board (REB). A total of 59 patients, with 65 lesions, were treated during this period, and included in the analysis. A total of seven of these lesions were treated with a palliative intent, and were included. While the majority of the lesions (64, or 98%) were malignancies, there was one patient who received post-resection radiotherapy for prevention of keloid scar formation. The majority were BCC (54% or n = 35) or SCC (31%, n = 20), though melanoma and melanoma-in situ, Merkel cell carcinoma and cutaneous manifestations of non-Hodgkin lymphoma (NHL) were also seen. Median age at the time of diagnosis was 82 with a range of 45 to 97 years. Additional demographic details of the patients are presented in Table 1. The most common fractionation scheme was 40 Gray (Gy) in 10 fractions given daily (48.2%, n = 28), though a range of doses were used, from 15 Gy/3 to 60 Gy/30 fractions.

**Table 1:** Summary of patient demographics. BCC: Basal cell carcinoma; SCC: Squamous cell carcinoma.

Patient Demographics	No. (%)
Sex	
Male	32/59 patients (54%)
Female	33/59 (56%)
Age	
Median	82 years
Range	45–97 years
Location	
Nose	15/65 lesions (23%)
Scalp	14/65 (22%)
Cheek/Lip	11/65 (17%)
Forehead/temple	19/65 (30%)
Misc	6/65 (9%)
Histology	
BCC	35/65 lesions (54%)
SCC	20/65 (31%)
Keloid scar	1/65 (2%)
Lymphomas	3/65 (5%)
Melanoma	2/65 (3%)
Lentigo Maligna	3/65 (5%)
Merkel Cell Carcinoma	1/65 (2%)
Follow up	
Median	8 months
Range	4.1–11.9 months

**Figure 1:**
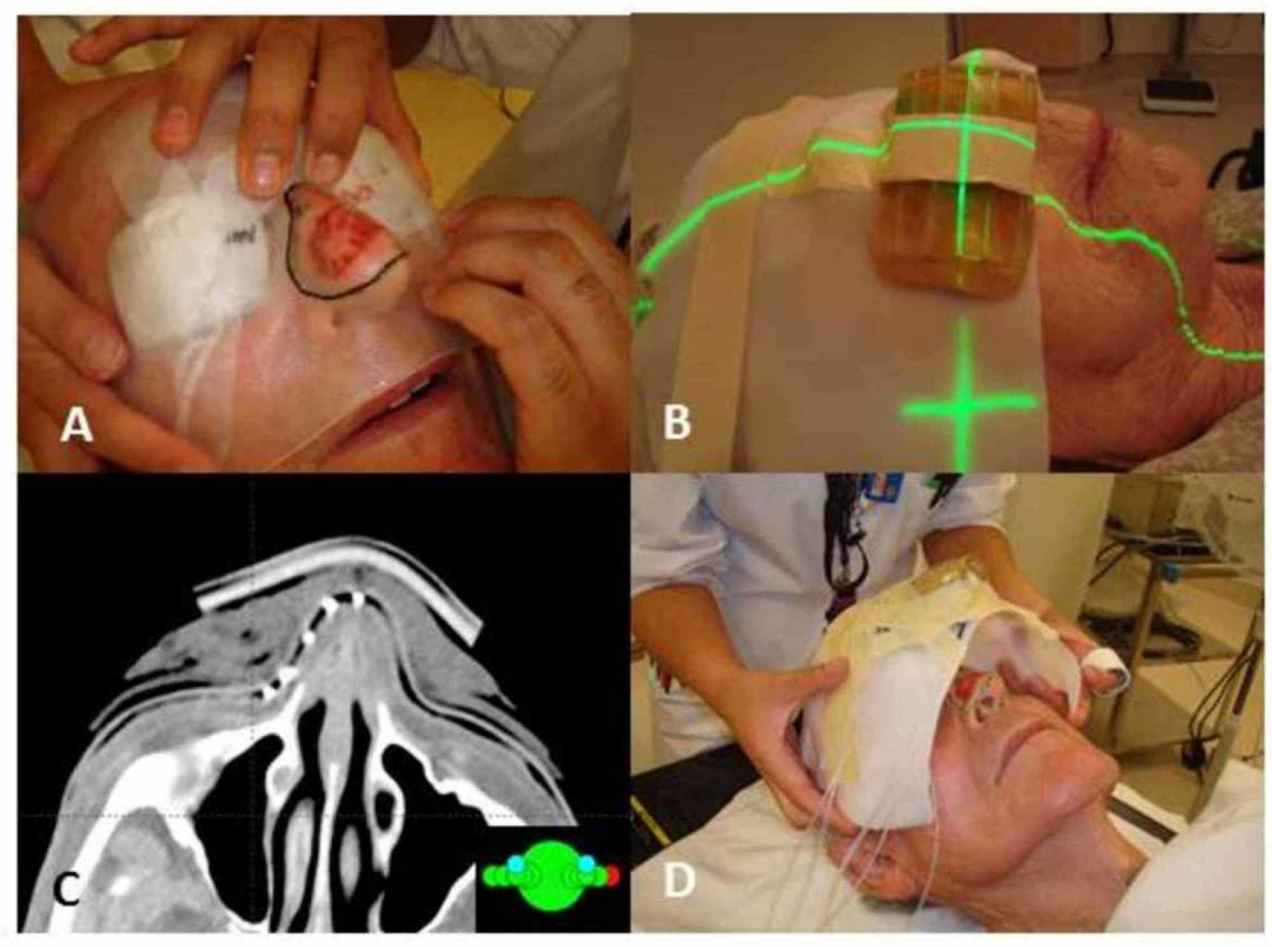
Localization of the target volume by the treating radiation oncologist (A), a patient with the surface mould in place at the time of the CT simulation scan (B), a CT image of a patient with the localizing radio-opaque wires and surface mould in place (C), and a surface mould with hollow catheters in place being placed on a patient prior to a brachytherapy session (D).

All patients underwent CT-based planning. The target volume was marked clinically by the oncologist using a radio-opaque wire (Figure 1A). Individualized surface moulds were designed for each patient (Figure 1B, 1C). The surface moulds used entailed supraflab containing channels for the brachytherapy catheters - typically spaced 1 cm apart - adhered to an Aquaplast mould shaped specifically to the patient’s external contours in the area to be treated (Figure 1D). The treatments were planned using the BrachyVision planning software (Varian Medical System, Palo Alto, CA). HDR brachytherapy was delivered using a 192Ir source.

Treatment was prescribed at a depth of 0.5 cm and the plan was optimized with the intent that the wired target was covered by 90% of the dose. Based on the radio-opaque wires placed by the treating oncologist, at the time of the planning CT scan, contours for the target volume were retrospectively segmented on the axial CT images. This was defined as the clinical target volume (CTV) or the target volume (TV). No expansion for planning target volume (PTV) was generated or used. The median target volume was 12.85 cc (range: 0.63-130.1 cc). The median depth of the 100% isodose line (IDL) was 0.5 cm from the surface. The median dose at the skin surface was 126.5%. The median V90, V95 and V100 values (or, the volume of the target volume receiving 90%, 95% and 100% of the prescribed dose) were 92.3%, 84.6% and 73.7%, respectively. The overall median dose to the target volume was 108.9%.

## Results

**Figure 2:**
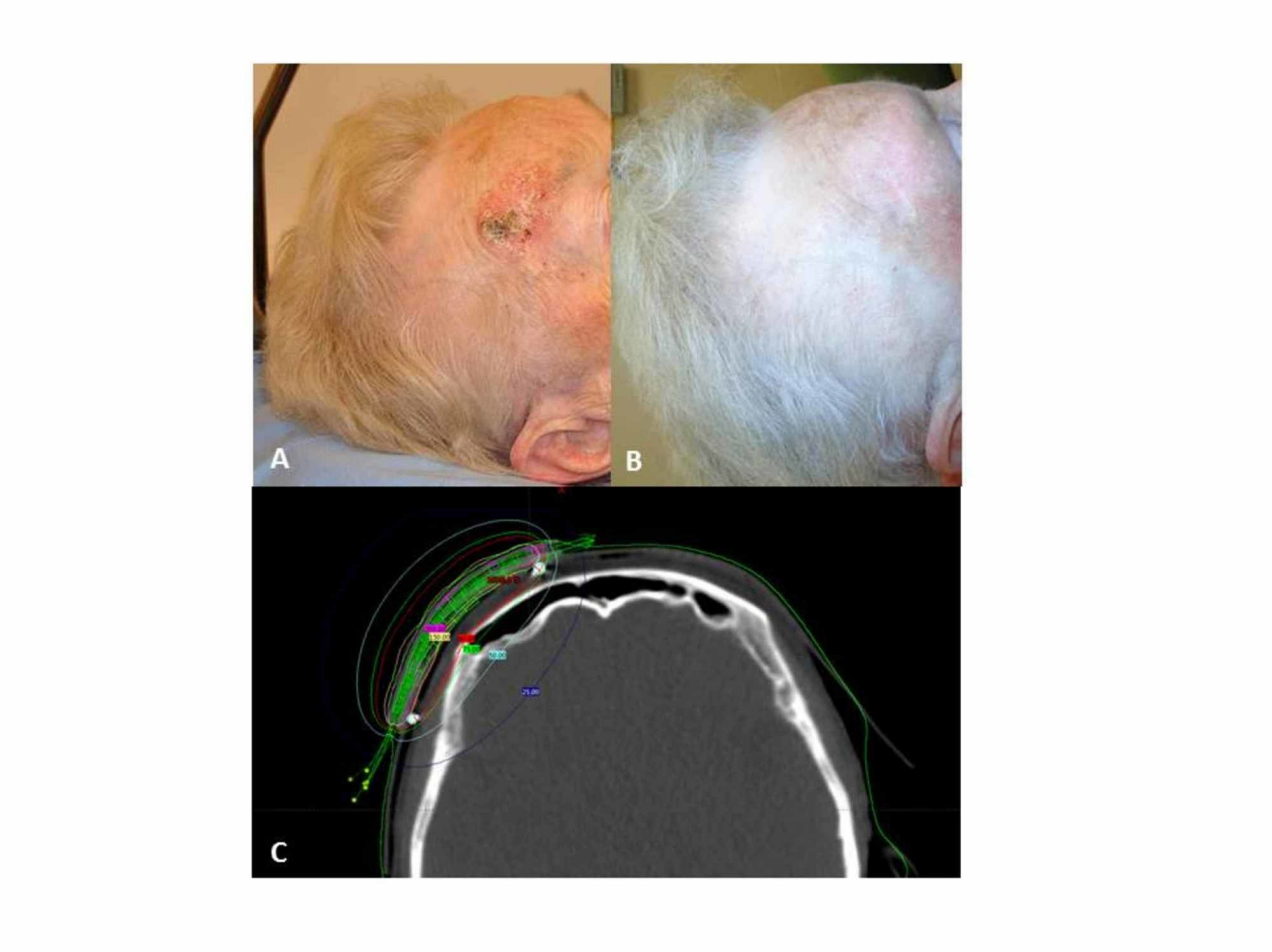
Photograph of a 91-year-old with squamous cell carcinoma (SCC) of the forehead before treatment with 42.5 Gy/10 fractions (A), two months after brachytherapy (B), with a CT image of the brachytherapy plan (C).

After a median follow-up of eight months, the two-year OS for the entire cohort was 77.6%. A total of 20 patients had died at the time of analysis. Only two had died due to progression of their skin malignancies (SCC and melanoma), four died of unrelated malignancies (lung cancer, esophageal cancer and hepatocellular carcinoma [HCC]), and the majority died of unrelated comorbidities: coronary artery disease (CAD), renal failure or chronic obstructive pulmonary disease (COPD). For the lesions treated with brachytherapy, the complete response (CR) rate was 96.8% (61 lesions). Partial response (PR) was noted for two lesions and response could not be assessed in two patients. On follow-up, local recurrences (LR) were noted in five patients. Of these, three had BCC of the nose, one had an SCC of the right forehead, and one patient treated for a skin lesion with palliative intent had local and distant progression. Distant disease developed in four patients - three died of their disease. The two-year local control rate was 84.9%, and the two-year progression-free survival (PFS) was 71.5%. Univariate analysis was done to examine any potential relationships between tumor-related factors, and the various dose-volume parameters in terms of their impact on local control rates. None of these factors were found to be statistically significant on the log-rank test. There was also no significant difference when patients treated palliatively were included or excluded from the analysis. The results are presented in Table 2. Figure 2 shows a patient with a malignancy prior to brachytherapy, with the subsequent response photographed at two months post-treatment, and a screen capture of the brachytherapy plan.

**Table 2:** Summary of results from univariate analysis.

Variable Analyzed		2-Year Local Control Rate (%)	p-value
V90	V90 less than 95%	76.9	0.85
	V90 greater than 95%	88.9	
Median dose to target volume	Median dose less than 100%	75	0.94
	Median dose greater than 100%	88.2	
Histology	Basal cell carcinoma	70	0.94
	Squamous cell carcinoma	88.2	

All 59 patients completed treatment, and overall, brachytherapy was well tolerated. Toxicity - both acute and late - was graded according to Common Terminology Criteria for Adverse Events (CTCAE) v. 4.03 based on review of patient records and clinical photographs taken during and 2-4 months after brachytherapy. The most common grade 1 toxicities were: dermatitis (58%, n = 38), pruritus (42%, n = 27), pain (23%, n = 15), fatigue (22%, n = 14), dry skin (12%, n = 8) and alopecia (12%, n = 8). For a more complete list of toxicities noted, please see Table 3. The only grade 2 acute toxicity noted was dermatitis (32%, n = 21) and ulceration (8%, n = 5). There was no grade 3, 4, 5 toxicity. Late toxicities noted were all grade 1 or 2, including one case of grade 2 necrosis (2%). Common grade 1 late toxicities included: hypopigmentation (15.4%, n = 10), fibrosis (8%, n = 5), mild erythema (7%, n = 4) and telangiectasias (7%, n = 4). Please see Table 3 for the complete list of late toxicities.

**Table 3:** Summary of acute and late toxicities for patients due to brachytherapy.

Toxicity	Grade 0	Grade 1	Grade 2
Acute			
Dermatitis	6 (9%)	38 (59%)	21 (32%)
Ulceration	53 (81%)	7 (11%)	5 (8%)
Oral Mucositis	62 (95%)	2 (3%)	1 (2%)
Pruritis	38 (59%)	27 (41%)	0
Pain	50 (77%)	15 (23%)	0
Fatigue	51 (78%)	14 (22%)	0
Dry Skin	57 (88%)	8 (12%)	0
Alopecia	57 (88%)	8 (12%)	0
Conjunctivitis	61 (94%)	4 (6%)	0
Epistaxis	61 (94%)	4 (6%)	0
Nasal Congestion	62 (95%)	3 (5%)	0
Tearing of Eyes	63 (97%)	2 (3%)	0
Dry Mouth	64 (98%)	1 (2%)	0
Infection	64 (98%)	1 (2%)	0
Late			
Necrosis	64 (98%)	0	1 (2%)
Hypopigmentation	55 (85%)	10 (15%)	0
Fibrosis	60 (92%)	5 (8%)	0
Telangiectasia	61 (94%)	4 (6%)	0
Erythema	61 (94%)	4 (6%)	0
Thinned skin (atrophy)	64 (98%)	1 (2%)	0
Dry Nose	64 (98%)	1 (2%)	0
Rhinorrhea	64 (98%)	1 (2%)	0
Tearing of Eye	64 (98%)	1 (2%)	0

## Discussion

Superficial brachytherapy is a useful non-invasive technique for treatment of skin malignancies, which has good local control rates as well as excellent cosmetic and functional outcomes. We reviewed our institutional experience with this technique and report CR rates of over 95%, and two-year local control of about 85%. We found no significant toxicities and treatment was well tolerated.

Surgery is often the primary treatment for NMSCs (non-melanoma skin cancers), but often this may not be the best option, due to tumor location (and thus, cosmetic outcomes) or patient comorbidities. Radiation therapy is a useful technique where patient status, comorbidities or tumor location precludes resection, but it can have an impact on adjacent normal structures, resulting in toxicity or cosmetic changes, located within the radiation field. There is evidence suggesting similar rates of local control, late toxicity and cosmetic effects with brachytherapy as compared with EBRT, and Delishaj et al. noted in their literature review that on average, patients had excellent cosmetic results in 62% of cases, good results in 26% and poor results in 5.5% [3, 6, 14, 18]. That said, many of the studies are small and often retrospective, hence GEC-ESTRO’s assigning a grade B for the technique (moderate/strong evidence for efficacy but with limited clinical benefit - generally recommended) [4]. The largest study was performed by Gauden et al.. They prospectively followed 200 patients with 236 skin lesions treated with 36 Gy in 12 fractions using surface brachytherapy. Patients were evaluated regularly for toxicity and cosmetic results, and the LC was 98% (232/236) with a median follow-up of seven months [14]. Four patients developed local recurrences and were salvaged with surgery [14]. (Of note, all had had radiotherapy for positive margins post-initial resection). Moderate toxicity was noted and 88% of the patients had good/excellent cosmesis, with hypopigmentation noted in 13 cases (5.5%) and no telangiectasias [14]. All patients completed therapy. The LC rates compared favorably with EBRT. Several other retrospective studies and case studies showed excellent results with adequate follow-up - LC rates varied from 83.3% to as much as 100% [8, 17, 20-23]. The papers reported variable levels of follow-up, cosmetic results and toxicities.

Late toxicities were generally grade 1-2: cosmetic results were considered good in most patients (though how many is uncertain) [4]. Guix et al. noted 10.3% (14/136) grade 1 ulceration and 14% (19/136) grade 2 erythema post-brachytherapy [6]. He also noted good or excellent cosmesis at three months post-treatment in 92% [6]. Svoboda et al. reported their 87 patients generally experienced grade 1-2 toxicities (Ex. 26 patients with moist desquamation and 32 with mild redness or dry desquamation) - no moderate or severe late toxicities were reported [18]. A literature review by Delishaj et al. noted a range of late toxicity rates - from 0.84% in Guix et al.’s paper to 54% in a paper by Skowronek et al. [3, 6, 23]. A recent paper published by Kalaghchi et al. of 60 patients treated radically or adjuvantly noted 6.7% rate of grade 3-4 acute toxicities at three months post-brachytherapy, and one patient with late grade 3-4 toxicity which resolved at two years after treatment completion [8]. Even in more difficult to treat regions, such as eyelids, hands or pinna, good LC and cosmetic and functional outcomes were noted by multiple authors [4, 13, 24].

The research available uses a range of dose and fractionation schemes, where the dose is prescribed to, and what volume expansions are used [4, 25]. These recommendations mention a wide variety of prescription doses which are successfully used, but generally recommend prescribing to 3-5 mm below the skin surface [4]. This variation in prescriptions and definition of target volumes is also seen in much of the published literature. Allan et al. prescribed 45 Gy in 8 fractions with disease covered by the 80% IDL [24]. Gauden et al. prescribed 36 Gy in 12 fractions and used gross tumor volume (GTV) plus 5-10 mm to generate a PTV [14]. Guix et al. prescribed 60-65 Gy in 33-36 fractions, calculating the dose at 5 mm depth from the skin surface [6]. Somanchi et al. prescribed 40-45 Gy in 8 fractions to the 80% IDL, at 1.5-3.5 mm below the skin surface for custom mould brachytherapy to SCCs of the hand [13]. Jumeau et al. used a PTV equal to their CTV for their customized brachytherapy plans, and prescribed on the IDL where V100 ≥ 95% [12]. Ouhib et al. advocated for an additional margin of 2 mm for malalignment, but noted that an expansion from CTV to PTV was controversial in brachytherapy [25].

At our institution, we have also used a variety of doses, though 40 Gy in 10 fractions was the most common dose fractionation (used in 48.2% of cases). All 59 of our patients completed treatment: 53 were treated radically and six were treated palliatively. The median age of our patients was 82, which certainly played a role in why surface brachytherapy was the chosen modality in these elderly patients. Our control rates compare favorably with other brachytherapy literature, which have noted LC rates anywhere from 83.3% to 100%, though some studies involve a small pool of patients [3, 4, 6, 8, 10, 12, 16-19]. This also compares favorably with EBRT LC rates of 87-100% [26-28], and 95-99% for Mohs [5, 7, 8], particularly when you consider that many patients were not suitable for surgery due to location, or patient performance status or comorbidities.

Toxicity from treatment was mostly grade 1, for both acute and late. The highest acute toxicities noted were grade 2 ulceration and dermatitis. There was no grade 3, 4 or 5 toxicities. The highest late toxicity noted was a single patient with grade 2 necrosis. The necrosis manifested approximately 10 months post-brachytherapy. Arguably, more incidents of late toxicity may have been noted with a longer follow-up: our median follow-up for this study was nine months. A significant proportion of our patients died (some shortly after treatment completed), which limits follow-up data. But, this also speaks to the importance of selecting a non-invasive technique which does not cause significant toxicity in elderly patients with a lower performance status. While the noted differences in LC rates with regards to the median dose to the target volume and V90 are interesting, the small sample size of our population unfortunately limits analysis, and results were not statistically significant.

## Conclusions

Surface mould brachytherapy is a safe, effective modality for treatment of skin malignancies or tumors. Brachytherapy was overall well tolerated, with no grade 3-5 acute or late toxicities. This treatment is a good alternative option for those patients unwilling or unable to undergo surgery for their skin malignancies.
